# Polyplacophoran Feeding Traces on Mediterranean Pliocene Sirenian Bones: Insights on the Role of Grazing Bioeroders in Shallow-Marine Vertebrate Falls

**DOI:** 10.3390/life13020327

**Published:** 2023-01-24

**Authors:** Alberto Collareta, Marco Merella, Simone Casati, Andrea Di Cencio, Chiara Tinelli, Giovanni Bianucci

**Affiliations:** 1 Dipartimento Scienze della Terra, Università di Pisa, Via S. Maria 53, 56126 Pisa, Italy; 2 Museo di Storia Naturale, Università di Pisa, Via Roma 79, 56011 Calci, Italy; 3 Gruppo Avis Mineralogia e Paleontologia Scandicci, Piazza Vittorio Veneto 1, 50018 Badia a Settimo, Italy; 4 Studio Tecnico Geologia e Paleontologia, Via Fratelli Rosselli 4, 50026 San Casciano Val di Pesa, Italy; 5 Istituto Comprensivo “Vasco Pratolini”, Via Guglielmo Marconi 11, 50018 Scandicci, Italy

**Keywords:** biostratinomy, chitons, ichnotaxonomy, *Metaxytherium subapenninum*, *Osteocallis leonardii* isp. nov., palaeoichnology, Pascichnia, *Radulichnus*, taphonomy, Zanclean

## Abstract

Chitons (Polyplacophora) include some of the most conspicuous bioeroders of the present-day shallow seas. Abundant palaeontological evidence for the feeding activity of ancient chitons is preserved in the form of radular traces that are usually found on invertebrate shells and hardgrounds. We report on widespread grazing traces occurring on partial skeletons of the extinct sirenian *Metaxytherium subapenninum* from the Lower Pliocene (Zanclean) of Arcille (Grosseto Province, Tuscany, Italy). These distinctive ichnofossils are described under the ichnotaxonomic name *Osteocallis leonardii* isp. nov. and interpreted as reflecting substrate scraping by polyplacophorans. A scrutiny of palaeontological literature reveals that similar traces occur on fossil vertebrates as old as the Upper Cretaceous, suggesting that bone has served as a substrate for chiton feeding for more than 66 million years. Whether these bone modifications reflect algal grazing, carrion scavenging or bone consumption remains unsure, but the first hypothesis appears to be the most parsimonious, as well as the most likely in light of the available actualistic data. As the role of bioerosion in controlling fossilization can hardly be overestimated, further research investigating how grazing organisms contribute to the biostratinomic processes affecting bone promises to disclose new information on how some marine vertebrates manage to become fossils.

## 1. Introduction

Polyplacophorans, also known as chitons, are slow-moving, bilaterally symmetrical, dorsoventrally flattened molluscs that characteristically display a dorsal series of eight articulated shell plates or valves [1]. Consisting of aragonite, these valves may be embedded, to varying extents, into a fleshy muscular girdle. Extant polyplacophorans species number more than 940 [2], most of which are found in the intertidal zone, dwelling on hard substrates, although some forms can be found down to 8000 m water depth [1].

Although some polyplacophorans (especially those taxa that inhabit deep-water environments) are known as detritivores and carnivores, most chitons feed by rasping epilithic (encrusting) and especially endolithic (boring) macro- and micro-algae from the rocks on which they live thanks to their extremely hard radula. Grazing of the substrate typically results in the removal of a thin layer of rock, which makes chitons some of the most conspicuous bioeroders of the intertidal zone [3,4].

Abundant palaeontological evidence for the feeding activity of ancient chitons is preserved in the form of trace fossils that usually occur on calcareous invertebrate shells and hardgrounds [5,6,7,8,9,10,11], and much more rarely on vertebrate bones [12]. The oldest such traces appear to date back to the Cretaceous [12], but discoveries of even older specimens may be anticipated considering the Cambrian (ca. 500 Ma) origin of polyplacophorans [13].

Here, we report on widespread grazing traces occurring on partial skeletons of the extinct dugongid sirenian *Metaxytherium subapenninum* from the Lower Pliocene (Zanclean) of Tuscany, central Italy. These distinctive ichnofossils are described under a new ichnotaxonomic name and interpreted as reflecting the feeding activity of polyplacophorans. The taphonomic and palaeobiological implications of these finds are then discussed in light of the relevant neontological and palaeontological literature.

## 2. Materials and Methods

### 2.1. Institutional Abbreviations

GAMPS—Museo Geopaleontologico “Gruppo AVIS Mineralogia e Paleontologia Scandicci,” Badia a Settimo, Scandicci, Florence Province, Italy; MSNUP—Museo di Storia Naturale dell’Università di Pisa, Calci, Pisa Province, Italy.

### 2.2. Specimen Preparation and Documentation

The trace fossils described herein are located on three sirenian skeletons (MSNUP I-15892, GAMPS 62M and GAMPS 63M) that were prepared by means of mechanical removal of the embedding sediment and subsequently stabilised with Paraloid B62. Photographs of these specimens were taken using a Nikon D5200 digital camera equipped with a Sigma 50 mm F2.8 macro lens. Measurements were obtained with a standard analogue calliper.

### 2.3. Stratigraphic and Palaeoecological Framework

**Figure 1:**
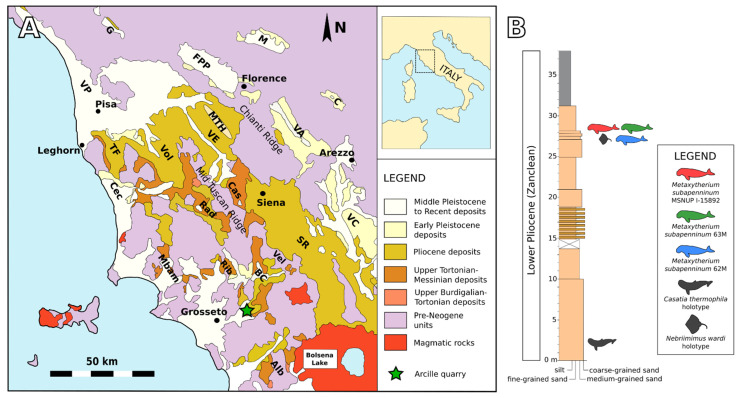
Geographic and stratigraphic setting. (**A**) Schematic geological map and distribution of the Miocene to Pleistocene basins of Tuscany. Alb, Albegna basin; Rib, Ribolla basin; Mbam, Montebamboli basin; BC, Baccinello –Cinigiano basin; Vel, Velona basin; Rad, Radicondoli basin; Cec, Cecina basin; TF, Tora–Fine basin; Vol, Volterra basin: VE, Valdelsa—lower Valdarno basin; SR, Siena–Radicofani basin; VC, Valdichiana basin; VA, upper Valdarno basin; FPP, Firenze–Prato–Pistoia basin; C, Casentino basin; M, Mugello basin; G, Garfagnana basin. The green star indicates the location of the study site (Arcille). Modified from Benvenuti et al. [20]. (**B**) Schematic stratigraphic section of the Lower Pliocene sedimentary succession exposed at Arcille, showing the position of the three trace-bearing sirenian skeletons that are studied herein (GAMPS 62M, GAMPS 63M and MSNUP I-15892) along with that of the holotypes of *Casatia thermophila* (a monodontid cetacean) and *Nebriimimus wardi* (a ?rajid skate). Modified from Bianucci et al. [21].

**Figure 2:**
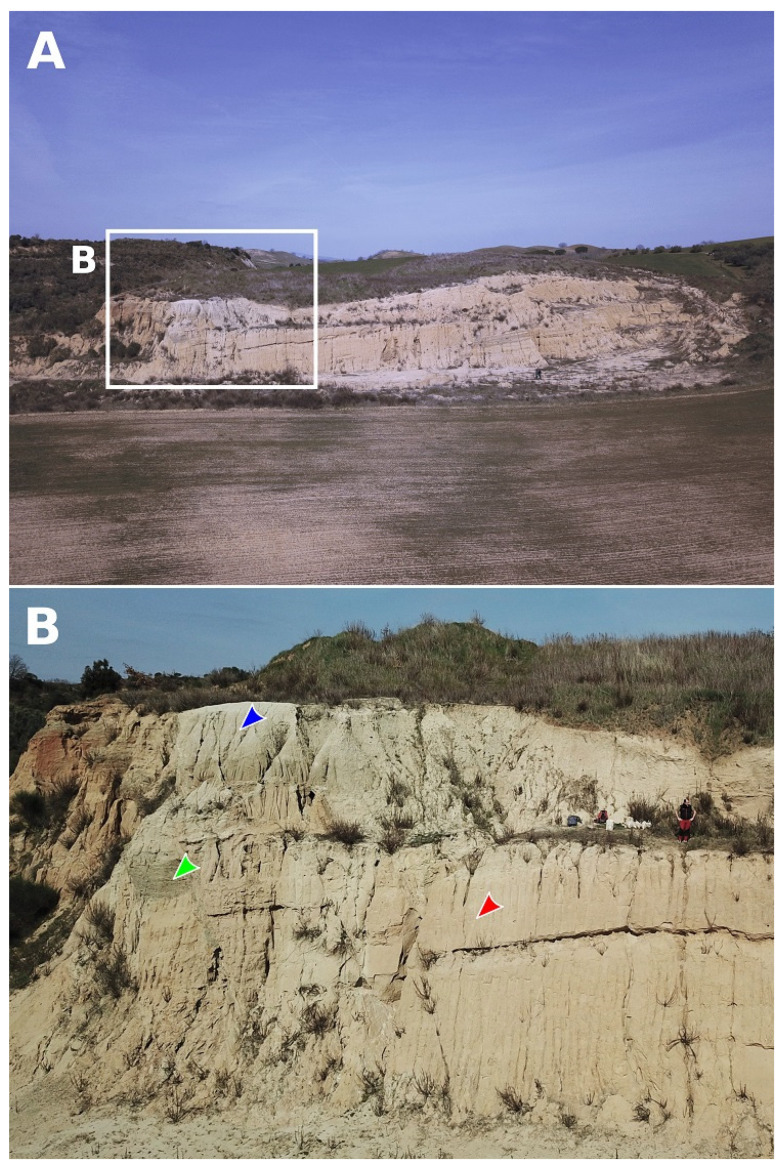
The Lower Pliocene sedimentary succession exposed at Arcille. Panoramic (**A**) and outcrop (**B**) views of the Arcille quarry. Note how the basal package of shoreface sandstones (red arrowhead) and conglomerates (green arrowhead) passes upwards to offshore mudstones (blue arrowhead). Geologist for scale in panel (**B**).

The fossil specimens dealt with herein were discovered at and around a sand quarry in the hinterland of Arcille (Campagnatico, Grosseto Province, Tuscany, central Italy). Arcille is located in the Baccinello–Cinigiano basin (Figure 1A), one of the post-collisional basins of the northern Apennines, whose Neogene infill comprises both continental and marine deposits [14]. The sedimentary succession cropping out at this site (Figure 1B) consists of terrigenous deposits dominated by yellowish, fossiliferous, shallow-marine shoreface sandstones with minor fluvial conglomeratic intercalations capped by greyish, open-shelf offshore mudstones [15,16] (Figure 2). These sediments have been referred by Dominici et al. [17] to their S2 Synthem, a lithologically diverse, Lower Pliocene depositional unit that includes fluvial conglomerates, fluvio-deltaic and shoreface sandstones, and shelf mudstones. Biostratigraphic analyses of the planktic foraminiferal assemblage from the mudstone division cropping out at Arcille indicate the lower part of the Zanclean, i.e., the Mediterranean Pliocene (=MPl) zone 2, which has been referred by Lourens et al. [18] to the 5.08–4.52 Ma time span [19].

Palaeontological highlights of the Arcille quarry include: (i) various specimens of *Metaxytherium subapenninum*, the latest sirenian of the Mediterranean Sea, which on the whole comprise a reference record for reconstructing the osteoanatomy, phylogenetic relationships and palaeoecological habits of this halitheriine dugongid species [15,19]; (ii) the holotype and referred specimen of *Casatia thermophila*, which represents one of the geologically oldest monodontid taxa, as well as the first and only representative of this odontocete family in the Mediterranean Basin [21,22]; (iii) the holotype and referred specimens of *Nebriimimus wardi*, an idiosyncratic rajiform batoid whose unusual multicuspid tooth morphology is currently unparalleled [23]; and (iv) some teeth assigned to the extant requiem shark species *Carcharhinus limbatus*, which represent the first occurrence of the blacktip shark as a fossil from both Europe and the Mediterranean Basin [24]. Other remarkable vertebrate fossils from the sandy strata exposed at Arcille include two partial skeletons of a marlin (cf. *Makaira* sp.), as well as abundant and diverse elasmobranch teeth and spines [19,23,25,26,27]. All things considered, the taxonomic composition of the marine vertebrate assemblage from Arcille indicates a warm-water, shallow-marine palaeoenvironment placed close to the coastline. In the same deposits, the remains of macro-invertebrates are also abundant, being dominated by bivalves (mainly pectinids and venerids, including the extinct large-sized clam *Pelecyora gigas*) with subordinate gastropods, scaphopods, echinoids and corals [25]. Given the presence of *P. gigas*, the molluscan assemblage can be referred to a stock of tropical or near-tropical taxa, categorised as the Mediterranean Pliocene Molluscan Unit (=MPMU) 1, whose most thermophilic members did not survive the cooling episode that affected the Mediterranean region around 3 Ma [28,29].

The three *M. subapenninum* specimens studied herein (GAMPS 62M, GAMPS 63M and MSNUP I-15892) originate from the highest portion of the sandstone division cropping out at Arcille. Such skeletons were discovered at two different horizons, resting upon as many shell beds [16,25]. The same stratigraphic intervals have yielded the holotype of *N. wardi* and the referred specimen of *C. thermophila*, as well as teeth of *C. limbatus* and fragmentary postcrania of cf. *Makaira* sp. [22,23,24]. The molluscan assemblage includes *Glycymeris nummaria*, *Limopsis aurita*, *Venus nux*, *Procardium indicum*, *Helminthia triplicata*, *Oligodia spirata*, *Thetystrombus coronatus* and *Neverita olla* [16]; scaphopods, barnacles and solitary corals (flabellids) are also present [25]. Macroscopic evidence of bioencrustation and bioerosion of the shell remains is apparently largely absent [25].

### 2.4. Nomenclatural Acts

The electronic edition of this article conforms to the requirements of the amended International Code of Zoological Nomenclature (ICZN), and hence the new name contained herein is available under that code from the electronic edition of this article. This published work and the nomenclatural acts it contains have been registered in ZooBank, the online registration system for the ICZN. The LSID for this publication is: urn:lsid:zoobank.org:pub:A89D692B-0408-4CFE-B59B-55F6BEBC8A49.

## 3. Systematic Ichnology

Ichnofamily Circolitidae Wisshak et al.,2019 [30]

Ichnogenus *Osteocallis* Roberts et al., 2007 [31]

**Type ichnospecies:***Osteocallis mandibulus* Roberts et al., 2007 [31]

**Other included ichnospecies:***Osteocallis infestans* Paes Neto et al., 2016 [32]; *Osteocallis leonardii* isp. nov., herein.

**Type horizon and locality:** Maevarano Formation, Upper Cretaceous of Madagascar.

**Ichnotaxonomic caveat:** Traces assigned by Roberts et al. [31] to *Osteocallis* are morphologically very close to those known under the ichnogeneric name *Radulichnus* [5]. In particular, Lopes and Pereira’s [11] emended diagnosis of *Radulichnus* may prove broad enough to arguably be used to include morphologies that have been referred to *Osteocallis*. However, many ichnologists accept that there are several substrate types (e.g., wood, bone, rock, unconsolidated sediment and even faecal matter) that are acceptable ichnotaxobases, i.e., useful to differentiate traces from an ichnotaxonomic point of view (Godfrey and Collareta [33], and the many references therein). At present, the ichnological community is nonetheless divided on what kind of substrates are ichnotaxonomically relevant, with some workers distinguishing between consolidated and unconsolidated substrates only (e.g., Donovan and Ewin [34]), and others taking a more differentiated approach (e.g., Zonneveld et al. [35]). Exemplary in this respect is the recent study by Höpner and Bertling [36], which on the one hand recognised that “*Radulichnus* on lithic substrates and *Osteocallis* on bone [are] well established despite their principally identical shape”, while on the other hand stating that “*Radulichnus* […] and other grazing traces should not be diagnosed based on substrate” because “the nutrition of grazers is not selective regarding its substrate”, to conclude that “the morphologically very similar *Osteocallis* may have been produced intentionally […], but this is highly speculative”. Thus, we acknowledge that a community consensus does not exist at present about the ichnogenus-level assignment of *Osteocallis* ispp., including the new ichnospecies described herein, which some workers may be willing to subsume into *Radulichnus*. That said, it is our contention that this new ichnospecies can be diagnosed against the closest representatives of both *Osteocallis* and *Radulichnus* sensu stricto, i.e., *O. mandibulus* and *R. transversus*, without recurring to substrate as an ichnotaxobase. Thus, even if future research may conclude that *Osteocallis* is a junior synonym of *Radulichnus*, in our opinion this would not affect the validity of the new ichnospecies described herein.

**Additional remarks:** As bioerosional features on bone, *Osteocallis* and *Radulichnus* may be referred to the recently described *Cubiculum* ichnofacies. Although the latter was originally restricted to nonmarine settings due to an apparent lack of studies on bone bioerosion in submarine environments compared to their subaerial counterparts [37], more recent research has seemingly interpreted the *Cubiculum* ichnofacies more inclusively [38].

*Osteocallis leonardii* isp. nov.

2003—*Radulichnus inopinatus*; Jagt [12], p. 176; pl. 1, Figures 1–4

2005—*Radulichnus*; Mulder et al. [39], p. 202; pl. 1, Figure 5

2013—*Radulichnus*; Janssen et al. [40], p. 156; Figure 8

2021—cf. *Radulichnus*; Bisconti et al. [41], p. 204; Figure 12a–c

Figures 3 and 5

**LSID**: urn:lsid:zoobank.org:act:B0016040-E16B-4E5A-8F51-0FEC870375D8

**Etymology**: Named after Giuseppe Leonardi, prominent palaeontologist and dean of Italian ichnology.

**Figure 3:**
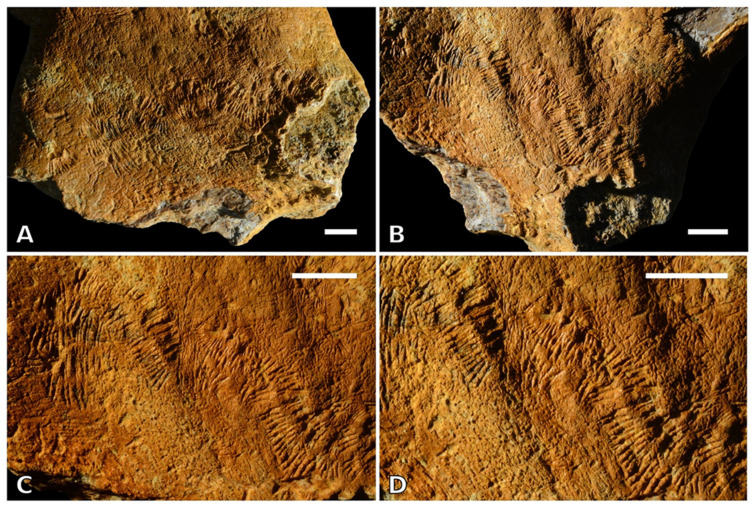
*Osteocallis leonardii* isp. nov., holotype, sculptured cortical bone area occurring on a fragmentary vertebra of the extinct dugongid sirenian *Metaxytherium subapenninum* (specimen MSNUP I-15892) at different magnifications and under different light conditions (**A**–**D**). Scale bars equal 5 mm in all panels.

**Figure 4:**
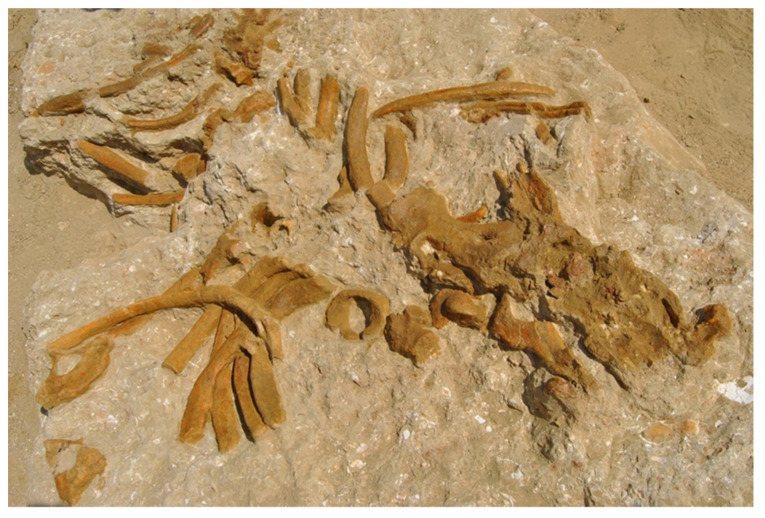
Partly excavated skeleton of the extinct dugongid sirenian *Metaxytherium subapenninum* (specimen MSNUP I-15892) at the Arcille quarry. The holotype and several other examples of *Osteocallis leonardii* isp. nov. occur on the external bone surface of this specimen.

**Holotype**: A sculptured cortical bone area (Figure 3) occurring on a fragmentary vertebra of the extinct dugongid sirenian *Metaxytherium subapenninum*. This vertebra is part of a disarticulated, almost complete skeleton collected in 2010 [15] and stored in the palaeontological collection of the MSNUP with accession number MSNUP I-15892 (Figure 4).

**Type locality:** A sunflower field 200 m north of the Arcille sand quarry (Campagnatico, Grosseto Province, Tuscany, central Italy; 42°47′18.16″ N, 11°17′01.05″ E).

**Type horizon:** Zanclean marine sandstones belonging to the S2 Synthem of the Tuscan Pliocene (see above for more details).

**Referred material from Arcille:** Besides the holotype, other well-preserved traces referred to *Osteocallis leonardii* isp. nov. occur on the supraoccipital, parietal and an anterior thoracic vertebra of MSNUP I-15892, as well as on at least twenty-seven fragmentary bones (mostly rib pieces) belonging to MSNUP I-15892 and two further skeletons of *Metaxytherium subapenninum* (GAMPS 62M and GAMPS 63M).

**Diagnosis:** Shallow trail of essentially straight or slightly arcuate furrows bored into external (cortical) bone surfaces. Furrows are organized in pairs side-by-side such that completely preserved trails appear as two parallel adjoining rows of closely parallel furrows. Groups of 2–3 appressed furrows are often observed along each row. *Osteocallis leonardii* isp. nov. may be comprised of a single trail or a network of randomly overlapping trails.

**Description and comparisons:** The holotype (Figure 3) consists of partly overlapping trails, each of which is between 4.5 and 5.0 mm wide and up to ca. 8.0 mm long. The single furrows are submillimetrical in depth, straight to slightly arched, and parallel to each other, thus forming two rows. The latter may or may not contact (or even slightly interdigitate with each other) at the midline. The spacing between the single furrows along each row is not greater than about one fourth the furrow length, and often distinctly smaller. Whereas most of the trails are sub-straight, one is unusually curved; curvature is reflected by the single furrows being arranged radially, those inside the arc being closer to each other (especially at their distal ends).

**Figure 5:**
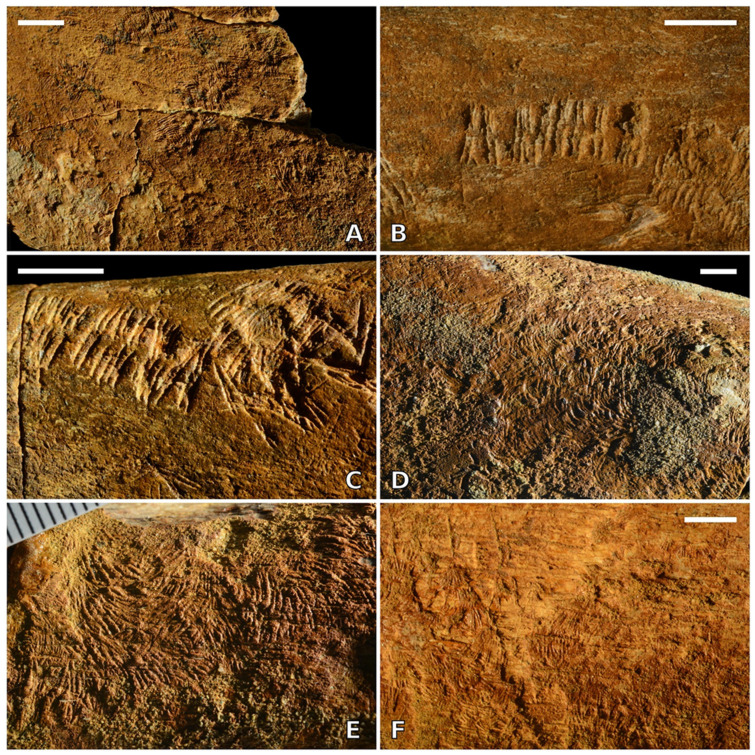
*Osteocallis leonardii* isp. nov., referred specimens from Arcille. (**A**) Small trails, less than 2.0 mm wide. (**B**,**C**) Trails in which the single furrows are arranged in relatively widely spaced groups of two or three along each row. (**D**–**F**) Cortical bone surfaces completely sculptured by dense, largely overlapping traces. Traces figured in panels (**A**) and (**C**–**F**) occur on the *Metaxytherium subapenninum* specimen MSNUP I-15892; traces figured in panel (**B**) occur on the *M. subapenninum* specimen GAMPS 63M. Scale bars equal 5 mm in panels (**A**–**D**,**F**); scale divisions in panel E equal 1 mm.

Trails from other bone fragments are up to more than 15 mm in length. The smallest individual trails that are recognizable as such are about 2.0 mm wide (Figure 5A). In some cases, the single furrows are arranged in relatively broadly spaced groups of two or three along each row (Figure 5B,C). On average, the smallest trails appear to be comprised of shallower furrows than the larger ones. Some bone surfaces appear as completely sculptured by dense, largely overlapping traces (Figure 5D–F). In that case, individual trails are generally no longer recognisable but locally, as well as for very short stretches only. Heavily grazed surfaces are often slightly but clearly depressed compared to the surrounding pristine bone cortex (Figure 5E,F). The trace may locally develop into deeper excavations, so that the single furrows are no longer discernible. Substrate topography may also influence the shape of the trace, with strongly convex surfaces resulting in trails formed by slightly asymmetrical furrow rows and/or in somewhat anomalous furrow outlines.

As already mentioned, it is our contention that *O. leonardii* isp. nov. can be diagnosed against the closest representatives of both *Osteocallis* and *Radulichnus* sensu stricto, i.e., *O. mandibulus* and *R. transversus*, based on trace morphology alone. In particular, *O. leonardii* isp. nov. differs from *O. mandibulus* by being comprised of overall less distinctly arched individual furrows that form straight (i.e., parallel), rather than C-shaped aligned pairs. Lopes and Pereira’s [11] definition of *R. transversus* describes the furrows as forming small clusters (numbering up to five individual incisions), as well as irregularly spaced, with interspaces equalling about one third to half the furrow length. This contrasts with the condition observed in *O. leonardii* isp. nov., in which the furrows are typically more closely (and often more regularly) spaced, as well as arranged in parallel adjoining rows. Actually, the holotype of *O. leonardii* isp. nov. (Figure 3) is morphologically closer to that of *O. mandibulus* (Roberts et al. [31]: Figure 4.1, 2) than to that of *R. transversus* (Lopes and Pereira [11]: Figure 3).

## 4. Discussion

### 4.1. Identification of the Tracemaker

*Osteocallis* was originally regarded as testifying to the incision of a bone substrate by the robust mouthparts of likely osteophagous insects [31]. Considering the submarine environment witnessed by the sediments that enclose the studied sirenian bones, insects are unlikely to be the producers of *Osteocallis leonardii* isp. nov. However, as already mentioned, traces similar to *Osteocallis* occur rather commonly on lithic substrates (including shells), wherein they are typically identified as belonging to *Radulichnus* ispp. and attributed to the grazing activity of molluscs provided with a hard radula (including gastropods and chitons) following the seminal work by Voigt et al. [5].

Deemed as “an exquisite example of nature at its best” [42], the chiton radula consists of a bilaterally symmetrical conveyor belt of continuously developing teeth arranged in two longitudinally elongated rows. Only the anteriormost teeth are effectively used for scraping the substrate; these dominant teeth are impregnated with crystals of magnetite that make them the hardest known mouthparts across all animals [43]. As the teeth converge toward the midline, they abrade the substrate to enable feeding on the embedded algae [44]. Thus, while feeding, chitons may leave sharp furrows that are oriented orthogonal to the longitudinal body axis to form elongated trails as the animals move forward to exploit new surfaces [5,6,10,11,12]. In contrast, grazing gastropods typically leave scars that are parallel to the longitudinal body axis, as well as arranged in fan-shaped or radiating patterns [6,45,46].

In light of these considerations*, Osteocallis leonardii* isp. nov. is regarded herein as the product of grazing by a polyplacophoran tracemaker. The dimensions of the largest *Osteocallis* traces that occur on *Metaxytherium* bones from the Pliocene of Arcille indicate that at least some of the producers would have been sizable. Furthermore, the relatively broad size range displayed by these ichnofossils suggests that both juvenile and full-grown chitons (or maybe different species thereof) grazed concomitantly on the *Metaxytherium* bones. Six polyplacophoran species belonging to Acanthochitonidae, Chitonidae, Ischnochitonidae and Lepidochitonidae are currently known from the Zanclean sediments of southern Tuscany, though not specifically from Arcille [16].

### 4.2. Palaeoethological Inferences

Traces testifying to the grazing activity of chitons have long been recognised in the fossil record, mostly occurring on bivalve shells. Like the radular scars left by many gastropods, putative chiton grazing traces on calcareous and lithic substrates conform to the ichnogenus *Radulichnus* [5] and have recently been assigned to the ichnospecies *Radulichnus transversus* [11]. Much more rarely, similar traces been described from vertebrate bones, often being identified as representatives of *Radulichnus* [12,39,40,41]. Regardless of the nature of the substrate, as well as of their ichnotaxonomic assignment, these ichnofossils have mostly been interpreted as evidence of the feeding activity of herbivorous chitons, specifically the grazing of epilithic and/or endolithic algae that developed on a bare substrate (including, in the case of vertebrates, defleshed bones) [5,6,7,8,9,10,11,12,47]. That said, feeding on residual soft tissues associated with invertebrate and vertebrate hardparts has also been proposed [11,12], especially in light of the observation that some chitons prefer to graze on the internal side of dead shells of bivalves. As a matter of fact, the deep-water polyplacophorans comprise carnivorous forms that consume sponges, bryozoans, molluscs, arthropods and foraminifera [48,49,50], and some active predators are also known among the shallow-water chitons [51].

The discovery of abundant polyplacophoran grazing traces occurring on cranial and postcranial bones of the extinct halitheriine dugongid sirenian *Metaxytherium subapenninum* from the Pliocene of Tuscany raises the question of what kind of feeding behaviour these ichnofossils testify to. In modern environments, chitons have sometimes been found in association with submerged mammalian carcasses in shallow-water actuotaphonomic studies [52], at times being regarded as transient species [53]. As regards the actual traces left by chitons on extant vertebrate hardparts, there is little neontological work on this topic [54]. A notable exception was recently provided by Higgs and Pokines [55], who observed some chitons grazing an algal mat grown on a whale bone sample, noticed the slight incisions that were left in the making, and concluded that chitons “[...] use the radula to scrape away or rasp algae that are embedded in the bone, thereby removing layers of bone as they do so”. Algal growth on bare bone is in fact well-known from many present-day marine, as well as nonmarine, settings [53,55,56,57,58]. All things considered, these observations are consistent with an algal browsing explanation for our Tuscan Pliocene traces.

That said, alternative hypotheses should be taken into account. One such hypothesis is suggested by the observation that several species of chitons in the family Mopaliidae (e.g., the mossy chiton *Mopalia muscosa*) eat meat (raw seafood) if it is offered in aquaria and may be partial scavengers [59]. Therefore, scavenging of the remaining integument and/or periosteum of the dead sirenian does also represent a reasonable possibility. Furthermore, and perhaps more surprisingly, the deep-water polyplacophoran species *Tripoplax balaenophila*, originally described as *Lepidozona balaenophila*, is known from a single locality off the Chilean coast by individuals that were found attached to skeletal remains (mostly fragments of whale ribs) and rocks at 240 m water depth, just beneath the shelf break [60]. Similar to the gastropod *Rubyspira osteovora* [61], *T. balaenophila* may represent a specialized member of the so-called “whale fall communities”, one that possibly feeds on whale bone [62]. As some typical members of the present-day, deep-water whale fall communities, including the osteophagous polychaete worm *Osedax*, can occasionally thrive on shallow-water carcasses [63,64,65], the *Osteocallis* traces recorded on the Pliocene *Metaxytherium* bones from Arcille may reflect osteophagy by specialized polyplacophoran scavengers. Supporting this interpretation, *Osteocallis* has been proposed to represent the product of feeding by osteophagous insects when occurring on bones deposited in nonmarine settings [31,32,66]. There are, however, significant concerns with this interpretation. First and foremost, only some select areas of the sirenian skeletons were subject to grazing, thus suggesting that the feeding chitons targeted precise locations on the exterior of the bones. In addition, to our knowledge, radular traces consistent with those left by chitons are not known from fossil deep-water whale fall communities, whereas those that have been published thus far from fossil bones do invariably originate from nearshore or shelf deposits.

In summary, algal grazing, carrion scavenging and bone consumption are all possible explanations for the widespread occurrence of polyplacophoran grazing traces on *Metaxytherium* bones from the Pliocene of Arcille, but the first hypothesis appears as the most parsimonious, as well as the most likely, in light of the available actualistic data. In any case, such traces testify to a rather long biostratinomic history for the Arcille sirenians, indicating that their bones were already bare (or almost so) and had undergone significant bioerosion at the time of the eventual burial.

### 4.3. Broader Palaeobiological Outcome

In addition to our Tuscan Pliocene material, scars that may conform to polyplacophoran grazing traces have thus far been reported from: (i) a mosasaur coracoid from the Upper Cretaceous of the Netherlands (as *Radulichnus inopinatus*; [12]); (ii) a hadrosaur tibia from the Upper Cretaceous of the Maastrichtian type area (as *Radulichnus*; [39]); (iii) a sea turtle carapace from the Upper Cretaceous of the Maastrichtian type area (as *Radulichnus*; [40]); and (iv) whale bones from the Pliocene of Piedmont, northern Italy (as cf. *Radulichnus*; [41]). Therefore, the present paper adds to the above short list by providing the first record of this kind of traces on sirenian bones. Despite having been generally identified as representatives of *Radulichnus*, all the aforementioned putative chiton traces that ornament vertebrate hardparts appear as morphologically close to our Tuscan Pliocene examples. Thus, they are reallocated herein to *Osteocallis leonardii* isp. nov. pending a comprehensive reappraisal of the genera *Radulichnus* and *Osteocallis*.

Bioerosion is a fundamental controlling agent of bone fossilization [67]. In marine (palaeo)environments, bone bioeroders range from bacteria and algae [56] to macro-invertebrates and vertebrates (Belaústegui [68], and the many references therein). Although the last twenty years have been characterized by an increasing interest in marine vertebrate taphonomy, most works on bioerosion deal with deep-water whale falls [68], though significant exceptions do exist [69]. Our scrutiny of palaeontological literature reveals that chitons have been active bioeroders of bone starting from the Upper Cretaceous at least. Given that chitons are known as prominent bioeroders of the present-day shallow seas and considering that these largely herbivorous molluscs would likely target bones that have already been weakened by the boring activity of endolithic algae, it is reasonable to hypothesise that the contribution of polyplacophorans to bone destruction in shallow-marine settings is—and long has been—more important than hitherto recognised. We expect the biostratinomic role of chitons as bioeroders of marine vertebrate skeletons to be less relevant for the highly pachyosteosclerotic sirenian bones than for those of, e.g., cetaceans, in which the somewhat protective external layer of compact cortical bone is usually distinctly thinner and bones are often less swollen [70].

## 5. Concluding Remarks

As already mentioned, chitons comprise some of the most conspicuous bioeroders of the present-day shallow seas. Our description of abundant grazing traces of Polyplacophora on skeletons of the extinct sirenian *Metaxytherium subapenninum* from the Pliocene of Tuscany indicates that chitons are also capable of significant bioerosion of bone. That similar traces occur on fossil marine vertebrates as old as the Upper Cretaceous reveals that bone has served as a substrate for chiton feeding for more than 66 million years. Whether these bone modifications reflect algal grazing, carrion scavenging or bone consumption remains uncertain, but the first hypothesis appears to be the most likely overall. Since the relevance of bioerosion as a major controller of vertebrate fossilization can hardly be overestimated, further research dealing with the contribution of chitons and other grazing organisms to the biostratinomic processes affecting bone promises to disclose new information on how some marine vertebrates manage to become fossils.

## Data Availability

Not applicable.
